# P-1470. RSV-Related Knowledge, Attitudes, and Practices Among US Primary Care and Specialist Physicians

**DOI:** 10.1093/ofid/ofaf695.1656

**Published:** 2026-01-11

**Authors:** Elizabeth M La, Kyli Gallington, David Singer, Zaneta Balantac, Noha S Eltoukhy, Yipin Han, Donald M Bushnell

**Affiliations:** GSK, Philadelphia, PA; Evidera, Wilmington, North Carolina; GSK, Philadelphia, PA; Evidera, Wilmington, North Carolina; GSK, Philadelphia, PA; Evidera, Wilmington, North Carolina; Evidera, Wilmington, North Carolina

## Abstract

**Background:**

During the 2024–2025 respiratory syncytial virus (RSV) season in the United States (US), RSV vaccination was recommended by the Advisory Committee on Immunization Practices (ACIP) for all adults aged ≥ 75 years and adults aged 60–74 years at increased risk for severe RSV disease. This study evaluated RSV-related knowledge, attitudes, and practices (KAP) among US healthcare professionals (HCPs), including primary care physicians (PCPs) and specialist physicians.

**Methods:**

A cross-sectional online survey of RSV-related KAP was administered to US HCPs between December 2024–January 2025. The survey targeted 700 HCPs, including PCPs (n=200), specialists (n=150), nurse practitioners and physician assistants (n=150), and pharmacists (n=200). Descriptive results are presented here on PCPs’ and specialists’ knowledge of adult RSV disease and vaccination, RSV-related attitudes, RSV vaccination practices, and potential vaccination barriers.

**Results:**

The final sample of 700 HCPs included 199 PCPs and 153 specialists (50 cardiologists, 50 endocrinologists, and 53 pulmonologists). Most PCP and specialist respondents reported being very familiar with RSV disease (83.9% and 80.4%, respectively). At the time of data collection, 81.3% of PCPs and 75.0% of specialists were aware that RSV vaccines were ACIP-recommended among all adults aged ≥ 75 years. However, fewer respondents were aware of the risk-based ACIP recommendation for adults aged 60–74 years (32.8% of PCPs and 27.0% of specialists). Most respondents perceived a benefit of having RSV vaccines available and recommended for all adults aged 60–74 years. Although more than 80% of PCPs and specialists reported recommending RSV vaccines to their eligible patients, these recommendations were not provided to all eligible patients consistently. Patient refusal or hesitancy was the most frequently reported barrier to recommending RSV vaccination to adults aged ≥ 60 years.

**Conclusion:**

Despite the familiarity of RSV disease among PCPs and specialists, additional efforts may be needed to address potential knowledge and practice gaps. These findings can help to inform HCP and patient education to support RSV vaccination among eligible patients.

Funding: GSK VEO-001082

**Disclosures:**

Elizabeth M. La, PhD, GSK: Employed by GSK|GSK: Stocks/Bonds (Public Company) Kyli Gallington, MPH, Evidera: Employed by Evidera|GSK: Employed by Evidera, which received funding from GSK to conduct this study David Singer, PharmD, MS, GSK: Employed by GSK|GSK: Stocks/Bonds (Public Company) Zaneta Balantac, ScB, Evidera: Employed by Evidera at the time of this study|GSK: Formerly employed by Evidera, which received funding from GSK to conduct this study Noha S. Eltoukhy, PharmD, MPH, GSK: Employed by GSK|GSK: Stocks/Bonds (Public Company) Yipin Han, MHS, Evidera: Employed by Evidera|GSK: Employed by Evidera, which received funding from GSK to conduct this study Donald M. Bushnell, MA, Evidera: Employed by Evidera|GSK: Employed by Evidera, which received funding from GSK to conduct this study

